# The mechanisms of hydrothermal deconstruction of lignocellulose: New insights from thermal–analytical and complementary studies

**DOI:** 10.1016/j.biortech.2011.06.044

**Published:** 2011-10

**Authors:** Roger Ibbett, Sanyasi Gaddipati, Scott Davies, Sandra Hill, Greg Tucker

**Affiliations:** BBSRC Sustainable Bioenergy Centre, University of Nottingham, Sutton Bonington Campus, Loughborough LE12 5RD, UK

**Keywords:** Bioethanol, Pretreatment, Hydrothermal, Mechanism, Lignocellulose

## Abstract

Differential Scanning Calorimetry, Dynamic Mechanical Thermal Analysis, gravimetric and chemical techniques have been used to study hydrothermal reactions of straw biomass. Exothermic degradation initiates above 195 °C, due to breakdown of the xylose ring from hemicellulose, which may be similar to reactions occurring during the early stage pyrolysis of dry biomass, though activated at lower temperature through water mediation. The temperature and magnitude of the exotherm reduce with increasing acid concentration, suggesting a reduction in activation energy and a change in the balance of reaction pathways. The presence of xylan oligomers in auto-catalytic hydrolysates is believed to be due to a low rate constant rather than a specific reaction mechanism. The loss of the lignin glass transition indicates that the lignin phase is reorganised under high temperature auto-catalytic conditions, but remains partially intact under lower temperature acid-catalytic conditions. This shows that lignin degradation reactions are activated thermally but are not effectively catalysed by aqueous acid.

## Graphical abstract

**Graphical abstract:**

**Figure:**
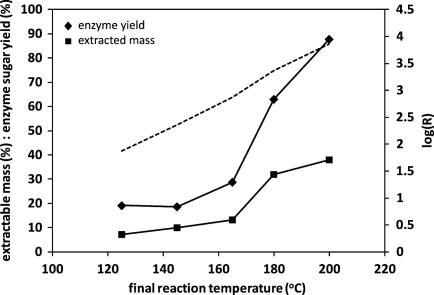

**Highlights:**

► Thermal analysis provides real-time data on hydrothermal reactions under realistic conditions. ► From DSC, hemicellulose hydrolysis has low enthalpy change but xylose degradation is exothermic. ► Hydrothermal exothermic degradation reactions may be similar to early stage biomass pyrolysis. ► DMTA shows that the polymeric structure of lignin in biomass is degraded at high temperatures. ► Hydrothermal reactions are effective at greater than 50% solids content.

## 1 Introduction

Second generation bioethanol processes are based on the fermentation of sugars derived from the cellulose and hemicellulose components of lignocellulosic plant materials. Considerable research and development activity has centred on the application of either auto- or acid-catalysed hydrothermal treatments for preliminary deconstruction of the plant cell wall (Kobayashi et al., 2011; Alvira et al., 2010; Talebnia et al., 2010). Such treatments are necessary to improve the enzyme digestibility of the lignocellulosic feedstock, to allow the intractable cellulose fraction to be converted to glucose sugars for fermentation to ethanol. Typical hydrothermal processing conditions result in the hydrolysis of the more accessible hemicellulose fraction, and the formation of soluble sugar monomers or oligomers (Thomsen et al., 2008). This is presumed to increase the porosity of the remaining cellulose rich residue, especially in the critical nanometre regime, allowing greater access by cellulose enzymes (Grethlein and Converse, 1991; Donaldson et al., 1988). Evidence from a number of studies has shown that the lignin phase is also disrupted under hydrothermal conditions, which must also impact on cellulose accessibility (Wörmeyer et al., 2011; Donohoe et al., 2008). However, there is need for a more complete understanding of the morphological reorganisations taking place during hydrothermal processing, to allow the development of fully predictive structure–reactivity models, which are able to account for the behaviour of the different cell wall components and their respective interactions (Acharjee et al., 2011; Kristensen et al., 2008). There is also a need for improved understanding of the complex chemical reactions taking place in the lignin and polysaccharide phases, which lead to the formation of acidic, furanic and phenolic compounds, which inhibit the subsequent fermentation of sugars by yeast organisms (Cantarella et al., 2004). Better structure–reactivity models will help to establish the optimum conditions for hydrothermal processing, maximising sugar yield whilst minimising inhibitor production. More robust models will also make it easier to identify new directions for investigation of more energy efficient approaches to biomass deconstruction.

In this study we have investigated the fundamentals of hydrothermal processing by applying thermo-analytical and complementary measurement techniques. In particular we have used high pressure differential scanning calorimetry (DSC) to obtain data under realistic moisture and temperature conditions. The technique of dynamic mechanical analysis (DMTA) has also been applied under novel controlled moisture conditions, which has provided information on the effect of processing on lignin phase transitions and structure. To aid interpretations we have compared data from two contrasting hydrothermal treatment protocols which have been used widely in other laboratory explorations. These are auto-catalysis in a high pressure tube reactor (Yang and Wyman, 2004), and dilute acid-catalysis using a laboratory autoclave (Silverstein et al., 2007). Of particular interest within these comparisons is the interplay between catalyst concentration and temperature as variables for optimisation.

## 2 Methods

### 2.1 Samples

Small quantities of wheat straw (Zebedee variety), comprising stem and leaf components, were knife milled to a 2 mm mesh size (P15 mill, Fritsch Gmbh) and were subjected to treatments in this form, or were further reduced using a rotary hammer-mill to 0.5 mm mesh size (LN3100, Perten Instruments AB). Some pre-treated samples were also subjected to the same hammer-mill size reduction. A reference eucalyptus pulp was supplied by Lenzing AG, containing 95% cellulose with residual 4% hemicellulose, according to the supplier’s assay. This was milled from sheet form using a domestic coffee grinder.

A hemicellulose reference sample was produced by extraction of a quantity of wheat straw in 4 M potassium hydroxide at room temperature, according to a modified literature method (Fang et al., 1999). The extract liquor was neutralised by addition of 6 M acetic acid, followed by precipitation of the hemicellulose by acetone addition. Further washing in water and ethanol was carried out, with final drying under vacuum at 60 °C. A reference sample of low sulphonate (kraft) lignin was supplied from Sigma Inc. (cat 471003: assay 4% sulphur).

### 2.2 Hydrothermal treatments

For auto-hydrothermal treatments, 2 g portions of as-received straw material were mixed with 8 ml of demineralised water and sealed into 1 in. diameter stainless-steel tube reactors. Loaded tubes tubes were held for 40 min in a preheated air-circulation oven, at different set temperatures. The heating time was noted from insertion in the oven, with the temperature following a ballistic heating profile, as measured separately for a non-volatile oil sample, via a thermocouple probe positioned through a cap with a hole. Knowledge of this profile allowed an estimation of the actual final sample temperature at each oven set temperature. After removal from the oven the tubes were quickly cooled under cold running water and the contents were then vacuum-filtered through glass microfibre filter paper (Whatman GF/A grade). The filter residue was washed with repeated amounts of demineralised water, then dried at 105 °C to constant weight, then cooled and reweighed. The ambient moisture content of the as-received straw was determined separately, for dry-basis calculation of the sample mass lost following treatment.

For comparative acid-catalysed hydrothermal treatments, 5 g quantities of straw were mixed in 500 ml glass Schott bottles with 200 ml of either 0.05, 0.1 or 0.2 M sulphuric acid. Bottles were loaded into a laboratory autoclave, programmed for 60 min heating time from ambient temperature up to 121 °C, in a saturated water vapour atmosphere, with a dwell of 30 min followed by cooling over 90 min to ambient temperature.

### 2.3 Differential scanning calorimetry

A Mettler–Toledo DSC 823 instrument was used for all measurements. Portions of dry samples were mixed with specific amounts of either water or dilute sulphuric acid by weight, with liquor contents expressed on a dry weight basis. Mixed samples were allowed to equilibrate in sealed containers and were then loaded and sealed into medium-pressure corrosion-resistant stainless steel DSC sample cells, with Viton O-ring seals. Thermal runs were carried out at a heating rate of 5 °C/min from −30 up to 220 °C, the upper temperature was chosen to coincide with the maximum safe working pressure of the sealed cells. An additional sample of untreated straw was loaded into an atmospheric aluminium pan, with a pierced lid. This was preheated to 100 °C to drive off ambient moisture, re-cooled and then heated at 5 °C/min up to 350 °C.

### 2.4 Dynamic mechanical thermal analysis

A Perkin Elmer DMA 8000 instrument was used for measurements. Hammer milled samples of as-received straw and dried treated residues were conditioned in the laboratory atmosphere to ambient moisture content, or were saturated with measured amounts of distilled water, followed by overnight equilibration. Portions of fully equilibrated samples were loaded into a stainless steel material pocket, which was folded closed and sealed at the edges with silicone oil, to avoid moisture evaporation on heating. The closed pocket was clamped in the DMTA instrument in single cantilever geometry, at 14 mm gauge length. Thermal runs were carried out from −30 to 130 °C, at a heating rate of 2 °C/min, at 1 Hz frequency, with a constant displacement amplitude of 50 μm. The pocket was not pressurisable and water vapour loss was seen typically at temperatures around 120 °C.

### 2.5 Biomass and hydrolysate composition

Analysis of soluble lignin in the hydrolysates was achieved by measurement of UV absorbance at 320 nm, according to the NREL method (Sluiter et al., 2008). Furanic compounds in the hydrolysates were determined by measurement of absorbance at 280 nm, subtracting the intensity at 320 nm, to account for spectral overlap with lignin (Martinez et al., 2000). Determination of organic acids was performed by high-pressure liquid chromatography using a Rezex ROA H+ organic acid column, at ambient temperature, with 0.005 N H_2_SO_4_ mobile phase at a flow rate of 0.5 ml/min, with UV detection. Full acid hydrolysis of straw, extracted hemicellulose and solid residues was carried out in 1 M sulphuric acid, for 2 h at 98 °C. Analysis of sugar monomers in all hydrolysates was determined by high-performance anion exchange chromatography with pulsed amperometric detection (Dionex, UK), using a CarboPac PA20 column with isocratic elution at a working flow rate of 0.5 ml/min. Glucose, xylose, arabinose, galactose were used as standards with mannitol as the internal standard. The proportion of oligomers in the hydrolysates was determined by comparison of sugar monomer compositions before and after a secondary full hydrolysis in sulphuric acid (Garrote et al., 2001). Analysis of lignin in straw and solid residues was carried out by extraction using acetyl bromide in water/dioxane solvent, followed by measurement of absorbance at 280 nm. Quantification was performed by calibration using the low sulphonate lignin reference material.

### 2.6 Enzyme assay

A solution of Celluclast® enzyme (Sigma Inc.: Product code C8546, Batch Number: 129K1359) was made up from dry powder in sodium citrate buffer (pH 5). This was predialised overnight against pure buffer solution to remove sugars and other low molecular weight impurities. Forty millilitres of enzyme solution were added to 100 ml screw-cap falcon tubes, into which were added dry 200 mg portions of untreated straw or treated straw residues, to give an enzyme activity of 40 FPU/g biomass, at 2 FPU/ml concentration. Enzyme digestions were carried out at 50 °C on an orbital shaker at 150 rpm over 24 h. Following digestion, the concentration of solubilised sugars was determined by the acid phenol method for analysis of reducing ends (Dubois et al., 1956). The percent total sugar yield was calculated by reference to the analysis of hydrolysates of fully acid hydrolysed samples by the same acid phenol method.

## 3 Results and discussion

### 3.1 Gravimetric and chemical analysis

**Fig. 1:**
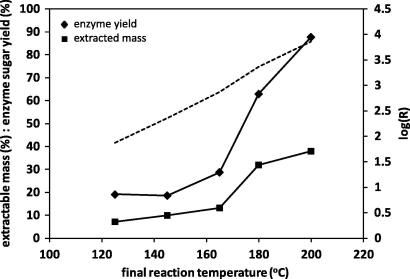
Hydrothermal reaction of straw at different oven set temperatures, in tube reactors at 4 ml/g liquor content, (■) extractable mass, (♦) enzyme digestibility of washed and dried residue. Also dashed line indicates severity factor (log *R*) at each reaction condition.

Fig. 1 shows that the extractable mass from straw increased as the temperature of the auto-catalysed reaction was raised. The inflection in the plot suggests that deconstruction reactions are only activated successfully above 170 °C, which corresponds with the steeper increase in subsequent enzyme digestibility of the residue. The measure of extractable mass has itself been used to determine the extent of hydrolysis type reactions, as the liberation of low molecular weight soluble material can be considered proportional to the number of random bond scissions within the biomass (Sharples, 1957). This principle allows the progress of reactions from contrasting acid and auto catalysis protocols to be compared on the same basis, without assumptions concerning Arrhenius or catalytic factors. From Table 1, auto-catalysis at 180 °C leads to a mass loss of 33%, resulting in an enzyme yield of 63%, where acid-catalysis at 121 °C in 0.05 M H_2_SO_4_ results in a similar mass loss, but an enzyme yield of only 43%. The development in digestibility with extent of reaction is clearly greater for the auto-catalysis protocol, where temperature is raised, compared to the acid-catalysis protocol, where acid concentration is raised but temperature is held constant at 121 °C. This in turn suggests that temperature is a key variable and some deconstruction reactions in the biomass may be thermally rather than acid activated.

Despite the differences in digestibility, the sugar monomer compositions of both auto and acid hydrolysates were similar, from Table 2, which indicates that in both cases it predominately the hemicellulose component which is extracted, with a minor proportion of lignin also becoming solubilised. This is reflected in the changes in compositions of the washed straw residues, shown in Table 3. However, from Table 2, the proportion of saccharide oligomers in the auto-catalysis hydrolysate is much higher than the acid hydrolysate, which has been suggested to be a result of differences in reaction mechanisms at a morphological level. However, as will be discussed these differences may be a reflection of different kinetics within the same overall reaction scheme.

For later discussions the reaction parameter of Overend and Chornet (1987) was also established for auto-hydrothermal reactions at different oven temperatures, with log(*R*) shown as a dashed line in Fig. 1, calculated by numerical integration over the ballistic heating profile, according to Eq. (1). Here *t* is reaction time and *T*_s_ is the sample temperature. For later results the effect of hydrogen ion concentration was accounted for by including a pH term in Eq. (2) for the combined reaction parameter, log(*M*), where *A* is, often taken as unity (Chum et al., 1990).

(1) $$\log R=\log t+\frac{T_{\text{s}}-100}{14.75}$$

(2) $$\log M=\log t+\frac{T_{\text{s}}-100}{14.75}-A.\;[\text{pH}]$$

### 3.2 Calorimetry of auto-hydrothermal reactions

Key examples of DSC thermograms are available in the E-Supplement to this paper. Dry straw measured in an unsealed pan gave rise to a progressive exotherm with an onset temperature around 240 °C, which has been associated previously with the pyrolysis reactions and volatile release from the hemicellulose component of lignocellulose (Yang et al., 2007). In contrast, the wetting of straw led to the development of a sharp exotherm with a significantly lower onset around 190–195 °C, which is evidence of the evolution of hydrothermal as opposed to dry thermal reactions of hemicellulose. The hydrothermal exotherm gained intensity with increasing water content, with the onset temperature remaining relatively constant, although the peak maximum was beyond the 220 °C limit of the sealed DSC cells. The evolution of the wet straw exotherm also confirms that hydrothermal reactions are activated at relatively high solids contents, with the intensity reaching an apparent maximum above 0.25 ml/g, which was borne out by results from further reactions of straw in the tube reactor. Table 1 shows that both extractable mass loss and corresponding enzyme yield were maintained up to 60% solids content (wet basis), following treatment for 40 min to a final temperature of 180 °C. This clearly has implications for the energy efficiency and water utilisation in a commercial process.

Wet straw also gave rise to an additional smaller exotherm around 150–175 °C, which shifted to lower temperature and became slightly larger with increasing water content, although this was not always observed repeatably. Possibly it is due to a more heterogeneous mixing event, as the cell wall matrix achieves sufficient thermal mobility to allow the ingress of water. An approximate integration of the peak gave an enthalpy around −20 J/g-dry weight, which is too small for a complete heat of wetting, which is approximately −130 J/g for cellulose (Mizutani et al., 1999). An endothermic event followed at a slightly higher temperature, around 160–180 °C, which was a precursor to the main exotherm, as has also been observed for materials undergoing pyrolysis (Milosavljevic et al., 1996). This is usually assigned to melting or liquefaction type reactions, where structural order is destroyed and entropy is raised, which could arise from either the hemicellulose or lignin phase. The event coincides with temperatures where a large increase was seen in extractable mass, from Fig. 1. The wet straw also gave rise to a minor endotherm around 50 °C, which is probably due to enthalpy relaxation of lignin at the glass-transition, as the material gains entropic freedom. This will be of relevance in the interpretation of dynamic mechanical data (Östberg et al., 1990).

The wetted reference hemicellulose also gave rise to a strong exotherm, with an onset around 185 °C, which supports the conclusion that the similar event in wet straw is due to hydrothermal breakdown of hemicellulose in the cell wall. The hemicellulose also exhibited the smaller mixing exotherm around 175 °C. In contrast, the wetted reference eucalyptus pulp did not give rise to any significant exothermic or endothermic response, which confirms that the exothermic event in wet straw is not due to reactions of cellulose. The lignin reference exhibited a small complex endotherm around 50–80 °C, which may again be associated with enthalpy relaxation, but there was no evidence of a hydrothermal exotherm at higher temperatures. However, it is appreciated this material has been chemically modified by the extraction process and will not fully represent the native state. As anticipated, a sample of previously reacted, washed and dried straw residue also no longer gave rise to a hydrothermal exotherm, as the majority of hemicellulose had been removed. This residue is rich in cellulose and also in lignin, from Table 3, although the lower temperature enthalpy relaxation assigned to lignin was less distinct. This is probably a result of irreversible reactions following initial treatment, which is apparent from the later dynamic mechanical data.

As discussed, the main hemicellulose exotherm probably has similar chemical origins to the exothermic event observed at higher temperatures following the pyrolysis of dry polysaccharides and sugars (Sheirs et al., 1998). These complex reactions are difficult to follow, ultimately resulting in the degradation of sugars to formic, acetic and other acids, water and carbon dioxide, and including formation of aromatic resinous chars (Demirba, 2000; Östberg et al., 1990). However, the dehydration of xylose to form furfural is an important intermediate reaction (Mansilla et al., 1998; Qian et al., 2005), which according to a simple calculation of bond energies has an enthalpy change around −800 J/g. This is in line with experimentally measured enthalpies for thermal degradation of dry polysaccharides under closed cell conditions (Raemy and Schweizer, 1983). This or related dehydration reactions may therefore account for at least a proportion of the main wet-state auto-hydrothermal exotherm.

The physical mobilisation or plasticisation of cell wall polymers by water will cause dehydration and other degradation reactions to be initiated at lower temperatures than under dry pyrolysis conditions. Water will also aid in the solvation of reactants or products, or may act as a co-reactant in the formation of activated intermediates (Qian et al., 2005). The precise balance of reaction pathways and associated kinetics will be influenced by the level of hydration of the lignocellulose cell wall and potentially any previous wet processing.

### 3.3 Calorimetry of acid-hydrothermal reactions

Liberation of acetic and formic acid during hydrothermal reactions will give rise to a small self-catalytic effect. However, the direct addition of protons from mineral acid will have a much greater influence on reaction rate, and potentially also on reaction mechanism. In this study a series of mixtures of straw were made up for DSC measurements, with 0.05, 0.1 or 0.2 M concentrations of sulphuric acid added to give a liquor content of 1 ml/g. This mirrored the range of acid concentrations used for the autoclave acid-catalysis protocol. From this investigation it was found that the hydrothermal exotherm shifted to lower temperature as acid concentration was increased, with the peak maximum found at about 212 °C for the straw mixture in 0.1 M acid, where it remained above the 220 °C limit for the mixture 0.05 M acid. However, the exotherm was also apparently reduced in intensity by the presence of acid, and in 0.2 M acid was completely suppressed.

The shift in the degradation exotherm to lower temperature is consistent with the expected reduction in activation energy due to acid catalysis. (Kunihisa and Ogawa, 1985). However, the apparent reduction in the magnitude of enthalpy change is more difficult to interpret, which is presumably a result of a modification of the balance of degradation pathways, so the cumulative enthalpy change is now close to neutral. Possibly the presence of acid induces early condensation reactions and the formation of a greater proportion of resinous material, reducing the reactant concentration available for later exothermic degradation. Alternatively, acid catalysis may induce extended hydrolysis reactions, which may be endothermic in nature. The shift of reactions to lower temperatures may increase the opportunity for physical solvation of small molecule products, which may also be an endothermic process due to entropy gains.

For comparison, a sample of the reference hemicellulose was mixed at 2 ml/g proportion with 0.2 M sulphuric acid. The exotherm peak maximum was again shifted below the 220 °C experiment limit, down to around 215 °C. The pure hemicellulose has much greater aqueous accessibility than the as-received straw, so the effective mole ratio of acid to biomass is lower. This may explain why the exotherm is still apparent at this liquor content, although it is not seen in a thermogram of straw mixed with 0.2 M sulphuric acid at 1 ml/g. The exothermic-mixing and endothermic-melting features are still apparent for the straw mixtures at the two lower acid concentrations, although these fluctuations become less distinct at the highest 0.2 M concentration. All acid–straw mixtures gave rise to the lower temperature lignin relaxation, as this is a result of the physical glass transition, which occurs below the onset of any irreversible modification of the lignin structure.

### 3.4 Dynamic mechanical transitions in auto- and acid-hydrothermal residues

**Fig. 2:**
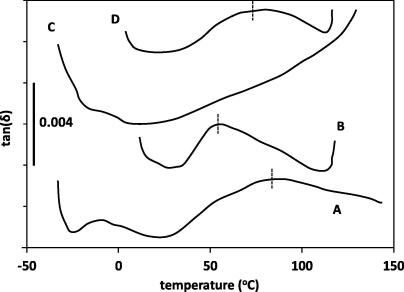
Variation in relative tan(*δ*) with temperature by dynamic mechanical thermal analysis, (A) for untreated milled straw at ambient atmospheric moisture, and (B) untreated milled straw in water saturated condition, (C) water saturated straw residue from auto-hydrothermal treatment at 200 °C, and (D) water saturated residue from autoclave acid-hydrothermal treatment with 0.1 M H_2_SO_4_ at 121 °C.

The DMTA technique is known to be sensitive to the lignin glass–rubber transition (Tg), which is indirectly responsible for the DSC enthalpy relaxation endotherm around 50 °C (Backman and Lindberg, 2001; Kelly et al., 1987). Clamping of whole straw stems in the DMTA instrument gave unreliable results, possibly because of the natural variability along the plant stem. Greater success was achieved with the measurement of hammer milled material, held in the steel material pocket. This method improved sampling homogeneity and also allowed for sealing of the sample against moisture loss. Fig. 2 shows the DMTA thermograms of untreated straw and treated residues, either dry conditioned at atmospheric moisture content, or in the water saturated state, following the mechanical loss-tangent (tan *δ*), which is a measure of the ratio of energy absorbed vs stored per cycle. The steel pocket contributes only to the continuous background, with the responses from the straw material superimposed. A broad tan(*δ*) peak was detectable in the atmospheric conditioned sample, which is associated with the lignin glass–rubber transition (Tg). The peak maximum was around 80 °C, which is similar to that reported for wood materials at equivalent moisture contents (Kelly et al., 1987). The peak shifted down to around 50 °C for a water saturated straw sample, suggesting a level of water plasticisation of the lignin matrix, which has also been seen in studies of wood.

In contrast, the lignin Tg peak was no longer detectable in the thermogram of the saturated auto-hydrothermal residue, in Fig. 2, despite the knowledge from the compositional assay that the majority of the original lignin is still present. This supports the evidence that the lignin has degraded to the point where it no longer has a polymeric phase structure, which according to visco-elasticity theory is a prerequisite for this mechanical transition. However, the tan(*δ*) thermogram of the water saturated acid-hydrothermal residue, also in Fig. 2, still showed evidence of a broad lignin Tg, with a tan(*δ*) maximum at around 75 °C. This is close to the position of the peak of the untreated sample at equilibrium atmospheric moisture, which suggests that in this case water plasticisation is less effective, which may indicate partial degradation of some of the aqueous accessible lignin structures by the acid-hydrothermal treatment. However, at least a proportion of the lignin phase would still seem to retain the polymeric characteristics existing in the original cell wall, presumably those structures with ether linkages more resistant to the aqueous acid. These DMTA comparisons indicate that lignin breakdown does occur thermally, but clearly requires higher temperatures than achieved in the laboratory autoclave. These results also confirm the significance of breakdown of lignin as a determining factor in achieving highest enzyme digestibility of lignocellulosic biomass by hydrothermal processing.

### 3.5 Auto and acid catalysed hydrolysis reactions

The necessary severity of reaction conditions required for auto-catalysed hydrolysis of hemicellulose leads to the formation of a range of degradation products which inhibit fermentation, including organic acids, phenolics and furans. Also, operation at high temperatures and vapour pressures places demands on the design of reactors and material handling, and on energy management and heat recovery. Operation at lower autoclave temperatures and pressures under acid catalytic conditions would therefore be attractive for these process considerations. However, theoretical severity factors will still be high, due to the inclusion of a pH term in Eq. (2). For the current autoclave protocol, an integration assuming linear heating and cooling gives a log severity factor of 1.2, at pH 1, holding for 30 min at 121 °C. The equivalent factor is −0.2 for auto catalysis using the tube-reactor, taking the data from Fig. 1 at a final sample temperature of 200 °C, assuming pH 4 as a result of weak acid formation in the reaction mixture. The high severity of the acid catalysed reaction is sufficient to hydrolyse the majority of the hemicellulose fraction, but unfortunately also leads to the secondary production of inhibitor byproducts. In addition, the required morphological deconstruction is incomplete at these lower temperatures, leading to only moderate enzyme digestibility.

The autoclave experiments confirm that acid-catalysed depolymerisation of hemicellulose is efficient at a 121 °C in 0.1 M H_2_SO_4_, although the lack of detectable heat-flow in this temperature range from the DSC data implies that the enthalpy of hydrolysis has a low value. In other studies the enthalpy of the hydrolysis of the β1–4 glucan bond of cellulose hydrolysed in aqueous mineral acid was estimated indirectly to be −4 J/g, superimposed on the larger exothermic degradation of glucose, of around −770 J/g (Kunihisa and Ogawa, 1985). The enthalpy of enzymatic hydrolysis of α and β1–4 glycosidic bonds of a range of disaccharides was found to between −24 and +35 J/g, using reaction calorimetry (Tewari and Goldberg, 1989), so hydrolysis of the β1–4 bond of xylan could equally result in a low enthalpy change. A simple consideration of bond energies suggests that the enthalpy of glycocidic bond hydrolysis will be zero, as the numbers of C–O and O–H bonds remain unchanged. From the tube reactor experiments auto-catalysed depolymerisation of hemicellulose is efficient above 170–180 °C, but is also difficult to detect by DSC. Under auto-hydrothermal conditions the hydrolysis reactions may now coincide with the smaller mixing and melting features which precede the main degradation exotherm, and hence may be associated with fragmentation and liquefaction type processes. It is tempting to suggest that some of these reactions are due to lignin as well as hemicellulose although the evidence from the DSC data is not conclusive.

### 3.6 The kinetics of oligomer formation

One interesting observation from this and previous studies is that that auto-hydrothermal reactions lead to the solubilisation of hemicellulose predominately as short-chain xylan oligomers (Garrote et al., 2001; Jacobsen and Wyman, 2000), where acid-hydrothermal treatment leads to a more complete hydrolysis to xylose monomers, as summarised in Table 2. Oligomer formation has been considered as an indicator of a specific physical mechanism of deconstruction at the morphological level, although other interpretations may be reasonable, based on a consideration of kinetic models for depolymerisation of polysaccharides (Sharples, 1957). The classical scheme for acid hydrolysis of polysaccharides involves proton attack at the glycocidic oxygen, followed by the formation of a resonance stabilised carbo-cation and the regeneration of the proton catalyst by reaction with water. For xylans in the straw cell wall, the progress of glycosidic scissions will cleave arabinose side groups and will also fragment the xylan backbone, with subsequent scissions reducing chain lengths till only xylose monomers remain. The reduction in degree of polymerisation (DP) has been modelled for a generalised linear polysaccharide, according to Eq. (3) derived from the original work of Ekenstam (Domvoglou et al., 2009). Here *P*_0_ and *P*_t_ are the number average DP at time zero and time *t*, and *k*_s_ is the rate constant for chain scission. The model assumes random scissions along polymer chains, with the inclusion of an accessibility fraction (*α*), which defines the proportion of glycosidic bonds in the polysaccharide available for reaction. This can be assumed to be 1 for hemicellulose in straw, as ultimately hydrolysis reactions are able to remove almost all pentose sugars from the cell wall. The model was originally developed to explore the hydrolysis of cellulose and so ignores the potential influence of covalent ferulic ester linkages between hemicellulose and lignin, which it is assumed will also undergo hydrolysis.

(3) $$P_{\text{t}}=1/\left[\alpha \left(1-\frac{1}{p_{\text{0}}}\right)\left(1-e^{-k_{\text{s}}t}\right)+\frac{1}{P_{\text{0}}}\right])$$

**Fig. 3:**
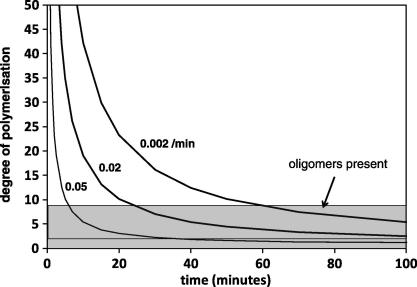
Model for polysaccharide depolymerisation with time, from Ekenstam equation (3), at different hypothetical rate constants, assuming full accessibility to hydrolytic agents. Initial number average DP = 250.

Hypothetical model outputs are shown in Fig. 3, with low, intermediate and high values for *k* selected to illustrate the range of kinetic profiles. The model shows the reduction in DP with time, to a point where the chain fragments are sufficiently small to become soluble in the reaction liquor, at a DP maybe of 6–10 (Sharples, 1957). The solubility of xylan fragments will also depend on presence or absence of side groups and/or residual ferulic ester linkages to lignin, but these may be considered as secondary factors. The important finding from the model is that a lower rate constant will result in the presence in the hydrolysate of a significant proportion of soluble oligomers at intermediate reaction times, which reflects the nature of the products observed under auto-hydrothermal conditions. In contrast, the autoclave acid-hydrothermal reaction proceeds with a higher rate constant and hence at equivalent reaction times a greater proportion of xylose monomers will be observed in the hydrolysate. The appearance of oligomers therefore does not in itself indicate a change in physical mechanism, implying that hydrolysis of hemicellulose within the cell wall follows the same mechanistic pathways both with and without acid catalyst.

## 4 Conclusions

DSC provides evidence that hemicellulose hydrolysis occurs with low enthalpy change, but xylose degradation reactions are exothermic and may be similar to those in the early stage of biomass pyrolysis, but activated through water mediation. The temperature and magnitude of the hemicellulose exotherm reduced with increasing acid concentration, suggesting a reduced activation energy and alteration in reaction pathways. The presence of xylan oligomers in auto-catalytic hydrolysates may be due to a reduced rate constant rather than a specific physical mechanism. DMTA confirms that lignin is reorganised at high temperature and that degradation reactions are activated thermally but are not effectively catalysed by acid.

## Figures and Tables

**Table 1:** Comparison of extractable mass and enzyme digestibility of hydrothermally treated straw.

	Conditions	Extractable mass (%)	Enzyme sugar yield from residue (%)
*Tube reactor (auto catalysed)*			
	Temperature (°C)		
Variable temperature (20% solids)	125	7.2	19
	145	10	19
	165	13	29
	180	32	63
	200	38	88
	Solids (%)		
Variable solids (180 °C)	20	34.5	82
	40	32.5	64
	60	30	67
	90	10	15
*Autoclave (acid catalysed)*			
	Concentration (*M*)		
Variable H_2_SO_4_ (constant 121 °C)	0	10	17
	0.05	33	43
	0.1	36	44
	0.2	39	50

**Table 2:** Chemical composition of typical auto- and acid-hydrolysate.

	Inhibitor concentrations in hydrolysate From 10 ml/g-straw reaction mixture (g/l)			Sugar proportions in hydrolysates (% total sugars)			
	Soluble lignin	Furans	Organic acids	Arabinose	Galactose	Glucose	Xylose
Auto-hydrolysate (ballistic heating to 200 °C)	2.6	0.5	5.1	10.0	2.9	11.6	75.1
Acid-hydrolysate (ramp to 121 °C in 0.1 M H_2_SO_4_)	2.0	0.15	4	11.0	3.5	11.7	73.7
Cold extracted hemicellulose				9.5	1.5	2.4	86.3

				Proportion of sugars as oligomers in as-received hydrolysate (%)^a^			
Auto-hydrolysate				0^b^	23	84	80
Acid-hydrolysate				0^b^	10	28	9

a Post hydrolysis conditions: 2 M H_2_SO_4_. 2 h @ 98 °C.

b Degraded by post-hydrolysis treatment.

**Table 3:** Typical compositions of straw and hydrothermal residues.

Material	Compositional assay (% total solid)			Sugar proportions in solid (% total sugars)			
	Cellulose	Lignin	Hemicellulose	Glucose	Xylose	Arabinose	Galactose
Straw (original)	39	20	28	64	31	4	1
Auto-hydrothermal residue (200 °C)	59	23	6	91	9	0	0
Acid-hydrothermal residue (0.1 M H_2_SO_4_, 121 °C)	58	24	2	97	3	0	0
