# Supplementary material for: The mechanisms of hydrothermal deconstruction of lignocellulose: New insights from thermal–analytical and complementary studies
Source: Bioresour Technol. 2011 Oct;102(19):9272–8. doi: 10.1016/j.biortech.2011.06.044 (PMC3268384; doi:10.1016/j.biortech.2011.06.044)
Supplement: Supplementary data 1 — Supplementary Figure [file mmc1.ppt]

## Slide 1
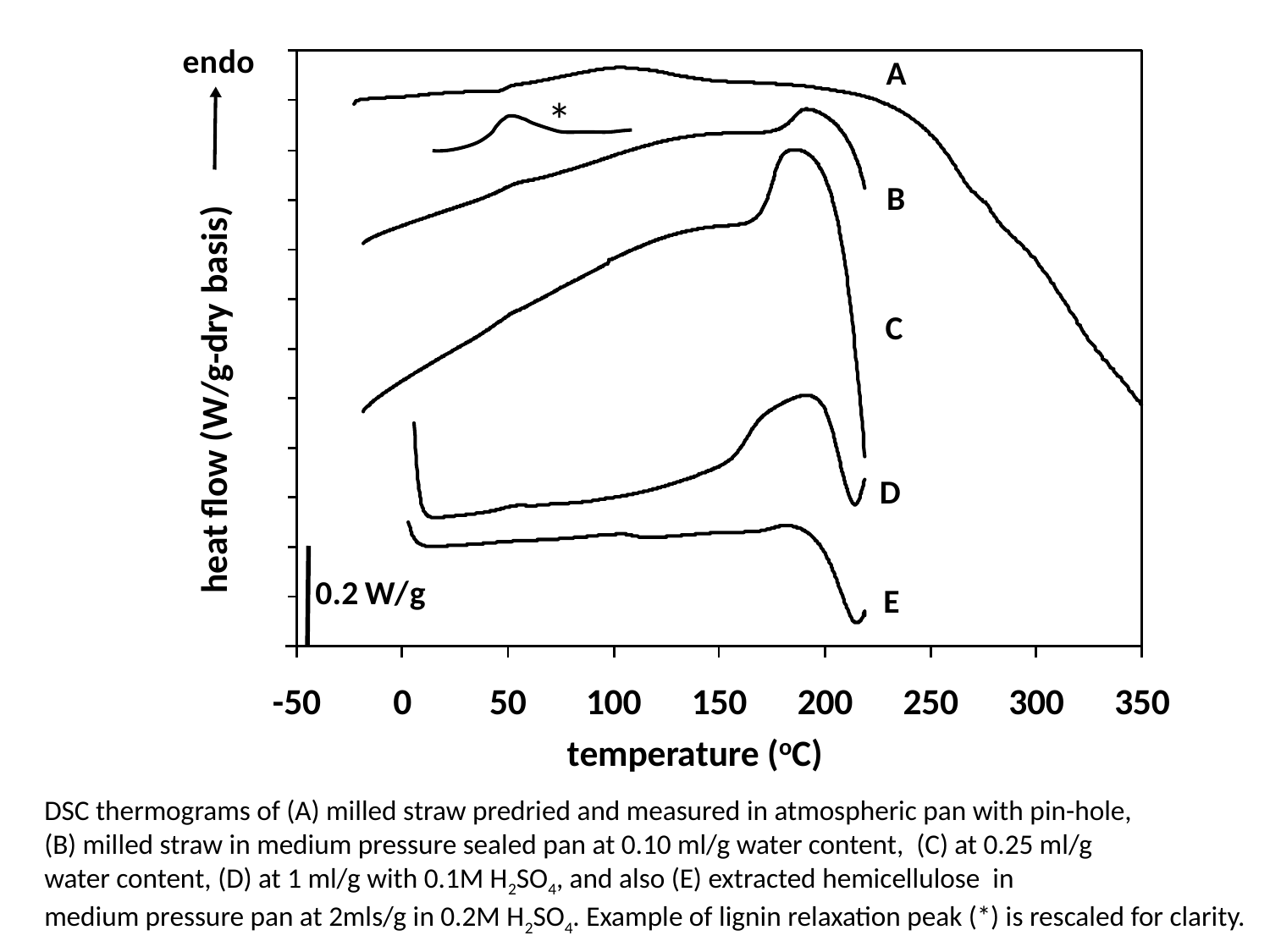

DSC thermograms of (A) milled straw predried and measured in atmospheric pan with pin-hole,
(B) milled straw in medium pressure sealed pan at 0.10 ml/g water content, (C) at 0.25 ml/g
water content, (D) at 1 ml/g with 0.1M H2SO4, and also (E) extracted hemicellulose in
medium pressure pan at 2mls/g in 0.2M H2SO4. Example of lignin relaxation peak (*) is rescaled for clarity.
